# Three-Dimensional Cartilage Regeneration Using Engineered Cartilage Gel With a 3D-Printed Polycaprolactone Framework

**DOI:** 10.3389/fbioe.2022.871508

**Published:** 2022-05-24

**Authors:** Gaoyang Wu, Lixing Lu, Zheng Ci, Yahui Wang, Runjie Shi, Guangdong Zhou, Shengli Li

**Affiliations:** ^1^ Department of Plastic and Reconstructive Surgery, Shanghai Ninth People’s Hospital, Shanghai Jiao Tong University School of Medicine, Shanghai, China; ^2^ Department of Otorhinolaryngology Head and Neck Surgery, Shanghai Ninth People’s Hospital, Shanghai Jiao Tong University School of Medicine, Shanghai, China; ^3^ National Tissue Engineering Center of China, Shanghai, China; ^4^ Research Institute of Plastic Surgery, Weifang Medical University, Weifang, China; ^5^ Shanghai Key Laboratory of Translational Medicine on Ear and Nose Diseases, Ear Institute Shanghai Jiao Tong University School of Medicine, Shanghai, China

**Keywords:** 3D cartilage regeneration, engineered cartilage gel, Polycaprolactone, inflammatory response, tissue engineering

## Abstract

The feasibility of the three-dimensional (3D) cartilage regeneration technology based on the “steel (framework)-reinforced concrete (engineered cartilage gel, ECG)” concept has been verified in large animals using a decalcified bone matrix (DBM) as the framework. However, the instability of the source, large sample variation, and lack of control over the 3D shape of DBM have greatly hindered clinical translation of this technology. To optimize cartilage regeneration using the ECG–framework model, the current study explores the feasibility of replacing the DBM framework with a 3D-printed polycaprolactone (PCL) framework. The PCL framework showed good biocompatibility with ECG and achieved a high ECG loading efficiency, similar to that of the DBM framework. Furthermore, PCL-ECG constructs caused a milder inflammatory response *in vivo* than that induced by DBM-ECG constructs, which was further supported by an *in vitro* macrophage activation experiment. Notably, the PCL-ECG constructs successfully regenerated mature cartilage and essentially maintained their original shape throughout 8 weeks of subcutaneous implantation. Quantitative analysis revealed that the GAG and total collagen contents of the regenerated cartilage in the PCL-ECG group were significantly higher than those in the DBM-ECG group. The results indicated that the 3D-printed PCL framework—a clinically approved biomaterial with multiple advantages including customizable shape design, mechanical strength control, and standardized production—can serve as an excellent framework for supporting the 3D cartilage regeneration of ECG. This provides a feasible novel strategy for the clinical translation of ECG-based 3D cartilage regeneration.

## 1 Introduction

The repair of craniofacial cartilage defect has always been a great clinical challenge (Raub et al., 2013; Xue et al., 2018), and recent development of tissue engineering technology has provided a promising strategy for repair and reconstruction of various cartilage defects (Makris et al., 2015; Kwon et al., 2019; Wei et al., 2021). By obtaining a small piece of autologous cartilage for chondrocyte isolation, followed by *in vitro* amplification, large-volume autologous cartilage regeneration can be achieved (Armiento et al., 2018; Wang et al., 2020; Liau et al., 2021). However, up to now, how to construct three-dimensional (3D) cartilage with specific shape and mechanical strength for the repair of craniofacial cartilage defects remains a huge challenge (Xia et al., 2018; Chae et al., 2021).

Various animal and clinical experiments have demonstrated that scaffold-free cartilage sheet technology can stably regenerate high-quality cartilage tissue *in vivo*. The injectable engineered cartilage gel (ECG) technology that derived from these examples has shown excellent performance for cartilage regeneration (unpublished data). To expand the scope of ECG technology application to the repair of craniofacial cartilage defects requiring a specific shape and strength (such as ear and nose reconstruction), our previous study proposed a novel 3D cartilage regeneration model based on the design concept of steel (framework)-reinforced concrete (ECG) and successfully regenerated homogeneous mature 3D cartilage in large animals using a decalcified bone matrix (DBM) as the framework (Ci et al., 2021). However, the main source of DBM is cadaveric donation, and the large diversity of donors, as well as of body sites from each donor, inevitably results in wide variation among DBM samples (Zhang et al., 2019). Moreover, the control of DBM morphology relies on manual cutting (Haghwerdi et al., 2021), which makes it difficult for it to be processed into particular shapes for the reconstruction of cartilage defects with complex 3D shapes (such as ear and nose) and thus greatly limits the clinical translation of regenerated cartilage based on DBM frameworks.

To further optimize the steel-reinforced concrete cartilage regeneration model, this study investigates the replacement of DBM with a 3D-printed polycaprolactone (PCL) framework to support 3D cartilage regeneration of the ECG. PCL is an FDA-approved biodegradable polyester material (Zou et al., 2015) with excellent biocompatibility (Arif et al., 2019; Yang et al., 2020; Lim et al., 2021) that can be prepared with a variety of 3D structures using customized design and 3D-printing technology (Li et al., 2020; Saracino et al., 2021). However, PCL has not yet been shown to be a suitable framework for the steel-reinforced concrete cartilage regeneration model. Therefore, to determine whether it is feasible to regenerate mature 3D cartilage using PCL-ECG constructs, the following key questions must be answered: first, does the PCL framework show good integration with ECG to achieve appropriate loading efficiency? Second, does the PCL framework trigger an inflammatory response that could interfere with cartilage regeneration? Third, do the PCL-ECG constructs successfully regenerate mature cartilage and retain their original shape in an immunocompetent large animal?

To answer the aforementioned questions, the current study explores the feasibility of 3D cartilage regeneration by combining a 3D-printed PCL framework with ECG, based on the previously established cartilage regeneration model. The characterization, biocompatibility, and inflammatory response of the PCL framework as well as the cartilage regeneration performance of the PCL-ECG constructs were systematically evaluated *in vitro* and in autologous large animals. The current study provides a feasible novel strategy for the clinical translation of ECG-based 3D cartilage regeneration for the repair of craniofacial cartilage defects.

## 2 Materials and Methods

### 2.1 Framework Preparation

DBM frameworks (Daqing Bio Co. Ltd., Chongqing, China) were cut into 7-mm-long, 5-mm-wide, and 2.5-mm-thick cuboid constructs. The PCL framework was fabricated using a 3D-printer. The properties of the PCL framework were designed using CAD and Mimics 17.0 software, and the frameworks were printed using a 3D layer-by-layer fused deposition modeling (FDM) printer (FoChif Tech HTS, China). In brief, PCL pellets (Mw 80,000, Sigma, United States) were melted (at 120°C) in a printing chamber and then printed with a lay down pattern of 0°/45°/90°/135° (top layer) or 0°/90° (bottom layer) along the z-axis. Thus, a double-layered PCL framework model was produced and then subsequently cut into cuboids with length, width, and height of 7, 5, and 2.5 mm, respectively. All frameworks were sterilized using ethylene oxide before use. The mechanical analysis of PCL and DBM framework was carried out using a mechanical testing machine (Instron-5542, Canton, MA, United States). All samples (*n* = 5 per group) were processed into a cuboid shape, and a constant compressive strain rate of 0.5 mm/min was applied until 80% of the maximum deformation. The stress and strain curves were obtained from the first 40%. The Young’s modulus was calculated from the stress and strain curves. The endotoxin content in the leach solutions of the frameworks were analyzed using a chromogenic endpoint Tachypleus amebocyte lysate (TAL) assay kit (Xiamen Houshiji, China), following the kit instructions as previously described (Wei et al., 2015).

### 2.2 Animals

A total of three 6-month-old goats (Shanghai Jiagan Biological Technology Co., Shanghai, China) were used in this study. All protocols of animal study were approved by the Animal Care and Experiment Committee of Shanghai Jiao Tong University School of Medicine.

### 2.3 Cell Culture

#### 2.3.1 Isolation and Culture of Goat Chondrocytes

After anesthetizing with 5% sodium pentobarbital (0.5 ml/kg), a slice of auricular cartilage (5 cm × 5 cm) was harvested from one ear of a goat and then dissected into 1-mm^3^ pieces, which were washed in phosphate-buffered saline (PBS) containing 1% penicillin–streptomycin (Gibco, Grand Island, NY, United States). The pieces were then treated with 0.15% collagenase II (Gibco) in Dulbecco’s modified Eagle medium (DMEM; Gibco) for 12 h at 37°C. Then, the isolated cells were collected and cultured in Dulbecco’s modified Eagle medium (Gibco BRL, Grand Island, New York, United States) containing 10% fetal bovine serum (Gibco BRL) and 1% antibiotic–antimycotic (Gibco BRL) T32U. Cells were passaged at >80% confluence. Chondrocytes in passage two or three were harvested to conduct the following experiments.

#### 2.3.2 RAW 264.7 Cells

Cells were cultured in Dulbecco’s modified Eagle medium (DMEM, Gibco BRL, Grand Island, NY, United States) containing 10% fetal bovine serum (Gibco BRL) and 1% antibiotic–antimycotic (Gibco BRL) and incubated in a humidified atmosphere of 95% air and 5% CO_2_ at 37°C. Cells were passaged at >80% confluence. Samples for fluorescent staining were seeded on 14-mm microscope cover glasses in a 24-well plate. To determine the inflammatory response of the frameworks, upon reaching 60–80% confluence, each group of RAW 263.7 cells was cultured in leach solution (supernatant from frameworks soaked in DMEM containing 10% fetal bovine serum for 72 h) and then cultured for 24, 48, and 72 h. In the positive control groups, 10 μg/ml lipopolysaccharide (LPS) was added to the culture medium, and a standard DMEM medium was used for the negative control.

### 2.4 Preparation of Engineered Cartilage Gel–Framework Constructs

Cartilage sheets were prepared as previously reported (Li et al., 2017). Furthermore, two- or three-passage goat chondrocytes were harvested, suspended, and then seeded in six-well cell culture plates at a density of 1.5 × 10^7^ cells/well. The chondrocytes were then cultured in a chondrogenic medium, containing 100 ng/ml IGF-I (R&D Systems Inc. Minneapolis, MN, United States), 10 ng/ml TGF-b1 (R&D Systems Inc. Minneapolis, MN, United States), 40 ng/ml dexamethasone (Sigma-Aldrich, St. Louis, MO, United States), 1% insulin–transferrin–selenium–linoleic acid (ITS, ScienCell, CA, United States), and 1% antibiotic–antimycotic (Gibco BRL) in DMEM for 5 days. The cartilage sheets were then minced into a gelatinous mass and collected in a syringe before being seeded in their respective frameworks to form constructs. The constructs were incubated for 2 h and then transferred into a 6-well plate containing the culture medium. After 3 days, the constructs were subcutaneously implanted in autologous goats. The cellular viability of the cartilage sheets and minced cartilage sheet (ECG) was evaluated using the Live/Dead Cell Viability Assay (Invitrogen, Carlsbad, CA, United States), following the manufacturer’s instructions, and examined by confocal microscopy (Nikon, Japan).

### 2.5 Biocompatibility of the Frameworks

#### 2.5.1 Scanning Electron Microscopy

The surface morphology and pore size distribution of the PCL and DBM frameworks were observed by SEM (Philips XL-30, Amsterdam, The Netherlands) at an accelerating voltage of 15 kV. The two types of ECG–framework constructs cultured for 24 and 72 h *in vitro* were washed with PBS and fixed overnight in 0.05% glutaraldehyde at 4°C. After dehydration in a graded ethanol series and critical point drying, the surface morphology and extracellular matrix (ECM) production of the constructs were observed by SEM.

#### 2.5.2 Engineered Cartilage Gel–Loading Rate

The ECG loading rate was determined from the ratio of the initial DNA content of the constructs and that 24 h after combination with ECG. The DNA content of the samples (*n* = 5 per group) was quantified using a Quant-iT PicoGreen dsDNA assay (Invitrogen, Carlsbad, CA, United States) as previously described (Chen et al., 2021).

#### 2.5.3 Live/Dead Cell Viability Assay

After 24, 48, and 72 h of culture in DBM and PCL leach solutions, the cellular viability of the cartilage sheets was evaluated using the Live/Dead Cell Viability Assay (Invitrogen, Carlsbad, CA, United States), following the manufacturer’s instructions, and examined by confocal microscopy (Nikon, Japan). Quantification of the ratio of dead cells to live cells was carried out using ImageJ and IHC Profiler Software (*n* = 5 per group).

### 2.6 Subcutaneous Implantation in Goats

After *in vitro* culture for 3 days, both constructs (PCL-ECG and DBM-ECG, *n* = 15 constructs per group in each goat) were subcutaneously implanted in autologous goats. As control groups, frameworks without ECG (DBM and PCL framework, *n* = 15 frameworks per group in each goat) and ECG without a framework were also implanted and injected into the goats, respectively. During surgery, each goat was anesthetized and endotracheally intubated. The constructs and frameworks were implanted in subcutaneous pockets made in the abdominal area. ECG without framework was injected using a syringe. The animals were allowed to recover from anesthesia after closure of the incisions. Samples were harvested at 1, 4, and 8 weeks postimplantation for gross, histological, immunohistochemical, and quantitative evaluation.

### 2.7 Inflammatory Response Evaluations

After 1 and 4 weeks of implantation, samples from all groups (*n* = 5 samples per group in each goat) were harvested for analysis of the inflammatory response. After gross observation, samples were fixed in 4% paraformaldehyde for 48 h, then embedded in paraffin, and sectioned into 5-mm-thick slices. Slices were stained with hematoxylin and eosin (HE). For immunohistochemical analysis, CD68 was detected using mouse anti-CD68 monoclonal antibody (ab955, 1:200, Abcam), followed by goat anti-mouse IgG H&L (HRP) (ab205719, 1:2000, Abcam). Apoptotic cells were detected by terminal deoxynucleotidyl transferase biotin-dUTP nick end labeling (TUNEL) using a TUNEL kit (Roche, Indianapolis, IN, United States), following the manufacturer’s instructions. The quantification of CD68 and TUNEL position area (%) was performed using ImageJ and IHC Profiler software (*n* = 5 per group).

### 2.8 Cell Morphology

For fluorescent staining, the cells were permeabilized with 0.1% Triton X-100 (Sigma-Aldrich) for 5 min at room temperature, washed with PBS, and then stained with DAPI and FITC–phalloidin. FITC and phalloidin (Sigma–Aldrich) were diluted in PBS in a 1:200 ratio and incubated on the samples away from light for 30 min. After incubation, the samples were washed with PBS three times. Cell nuclei were stained with DAPI for 8 min, after which the samples were washed with PBS three times. Imaging of RAW 264.7 cells was performed using a fluorescence confocal microscope (Nikon, Japan).

### 2.9 Enzyme Linked Immunosorbent Assay

Cell culture supernatant (1 ml) was collected after 24, 48, and 72 h of incubation and used for enzyme-linked immunosorbent assay (ELISA). Mouse IL-6 ELISA Kit, Mouse TNF-α ELISA Kit, and Mouse Cox-2 ELISA Kit (all ELISA kits; Invitrogen, Thermo Fisher Scientific, Waltham, MA, United States) were used according to the manufacturer’s instructions.

### 2.10 Quantitative Polymerase Chain Reaction

Gene expression of inflammatory cytokines was investigated using the real-time polymerase chain reaction (RT-PCR). The expression levels of *IL-6, TNF-α,* and *Cox-2* genes were analyzed. Each group of RAW 264.7 cells was collected, and the total RNA was extracted using the TRIzol reagent (Invitrogen), after which the total RNA was reverse transcribed using Moloney murine leukemia virus reverse transcriptase (Invitrogen). qPCR was performed using a Fast Synergy Brands Green Master Kit and a Light Cycler 480 system (Roche), following the manufacturer’s instructions. The forward and reverse primer sequences are listed in Table 1. The results were analyzed using the comparative threshold cycle method and normalized to the endogenous reference gene *β-actin*.

**TABLE 1:** Primers used in this study.

Gene	Primer
Mouse *TNF-a*	Forward: CCA CTC TGA CCC CTT TAC TC
	Reverse: GCC ATA ATC CCC TTT CTA AGT
Mouse *IL-6*	Forward: CGG AGA GGA GAC TTC ACA GAG
	Reverse: ATT TCC ACG ATT TCC CAG AG
Mouse *Cox-2*	Forward: TGG ATT CTA TGG TGA AAA CTG TA
	Reverse: TTG AAG TGG GTC AGG ATG TA
Mouse β*-actin*	Forward: CCT CTA TGC CAA CAC AGT
	Reverse: AGC CAC CAA TCC ACA CAG

### 2.11 Histological and Immunohistochemical Evaluations of Regenerative Tissues

After 8 weeks of culturing *in vivo*, samples of the PCL-ECG, DBM-ECG, and ECG groups (*n* = 5 samples per group in each goat) were carefully extracted from the goats. After gross observation and measurement, part of each sample (the rest of the sample was used for subsequent biochemical analysis) was fixed in 4% paraformaldehyde, embedded in paraffin, sectioned to give 5-mm thicknesses, and then mounted on glass slides for histological and immunohistochemical analyses. The slices were stained with H&E and safranin-O to evaluate the histological structure of the engineered cartilage (EC), and for the immunohistochemical analysis, expression of type II collagen (COL II) was evaluated to determine ECM deposition of the ECs using rabbit anti-collagen II polyclonal antibody (ab34712, 1:100, Abcam) with goat anti-rabbit IgG H&L (HRP) (ab205718, 1:2000, Abcam) as a secondary antibody. Quantification of the regenerated cartilage area (%) was performed using ImageJ and IHC Profiler software (*n* = 5 per group).

### 2.12 Quantitative Analysis

Quantitative analysis was performed as previously described (Jia et al., 2020). In brief, an electronic balance was used to measure the weight of all samples (*n* = 5 per group). The volume of each sample was measured using the water displacement method (*n* = 5 per group). The total glycosaminoglycan (GAG) content and the total collagen content of the samples (*n* = 5 per group) was quantified using the alcian blue method and hydroxyproline assay, respectively.

### 2.13 Statistical Analysis

Statistical analyses were performed using SPSS 23 (IBM, United States). Student’s t-test was performed to compare the mechanical properties and ECG loading efficiency of the frameworks. One-way ANOVA was performed to compare the results of the cytotoxicity evaluation of the frameworks and immunohistochemistry results. Two-way ANOVA was performed to test the interaction between two independent variables (time and material type for the quantitative analysis results, and time and leach solution type for the quantitative RT-PCR and ELISA results). Tukey’s honestly significant difference (HSD) *post hoc* tests were performed after ANOVA. Data are presented as the mean ± standard deviation (SD). The value of ∗*p* < 0.05 was considered statistically significant.

## 3 Results

### 3.1 Fabrication, Characterization, and Biocompatibility of the Frameworks

**FIGURE 1:**
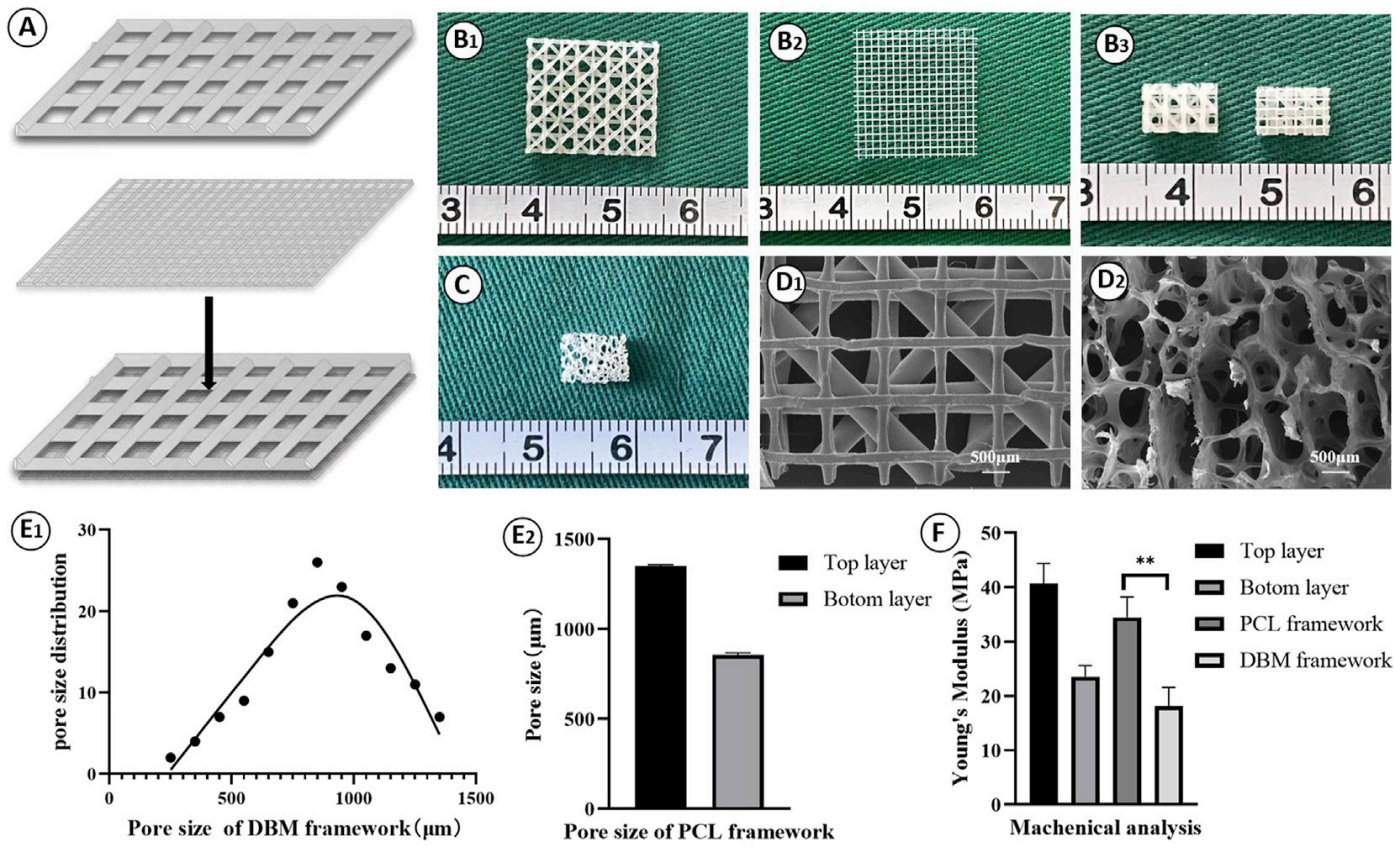
Fabrication and characterization of PCL framework: **(A)** The design of the 3D-printed PCL framework; Gross view of the top layer **(B1)**, the bottom layer **(B2)**, and the front and the back views **(B3)** for the 3D-printed PCL frameworks; **(C)** Gross view of the DBM framework; SEM images of PCL framework **(D1)** and DBM framework **(D2)**; Pore size of DBM framework **(E1)** and PCL framework **(E2)**; **(F)** Young’s modulus of the frameworks. Statistical significance: ∗∗*p* < 0.01.

Fabrication and characterization of the PCL framework was the first step of PCL-ECG construct preparation. As shown in Figure 1, a double-layered PCL framework structure was 3D-printed and assembled. The top layer with large pores was suitable for ECG loading, while the bottom layer with small pores was designed to prevent ECG loss (Figures 1A,B). Both SEM and pore size analysis revealed that the PCL framework had a uniform pore structure, while the DBM framework presented a clearly heterogeneous structure with varied pore sizes (Figures 1D,E), indicating the relative homogeneity and controllability of the PCL framework in terms of ECG distribution, mechanical properties, degradation rate, and shape maintenance. The mechanical analysis revealed that the mechanical strength of the PCL framework was significantly higher than that of the DBM framework (Figure 1F). The PCL framework had a mechanical strength close to that of native ear cartilage (Zhou et al., 2018), which may help it to maintain the original shape of the regenerated cartilage.

**FIGURE 2:**
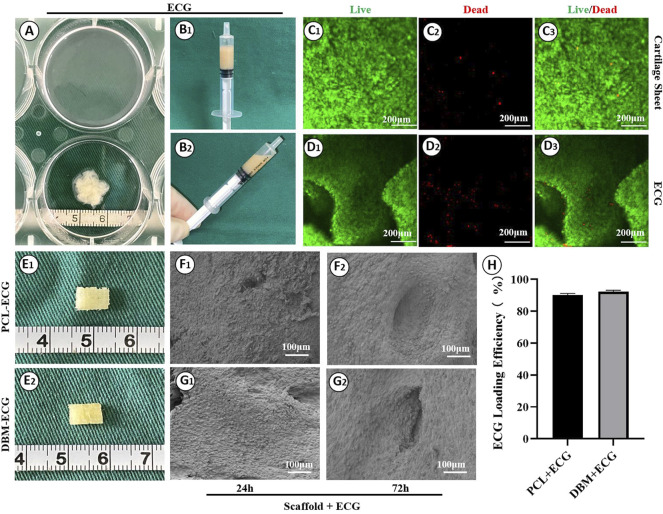
Preparation of engineered cartilage gel and its biocompatibility with the frameworks: Gross images of cartilage sheet **(A)** and ECG **(B1, B2)**; Live/dead staining of cartilage sheet **(C1–C3)** and ECG **(D1–D3)**; Gross images of the PCL-ECG **(E1)** and DBM-ECG **(E2)** constructs; SEM images of PCL-ECG **(F1, F2)** and DBM-ECG **(G1, G2)** constructs after culture in vitro for 24 h and 72 h; **(H)** ECG loading efficiency. Statistical significance: ns, no statistical significance.

**FIGURE 3:**
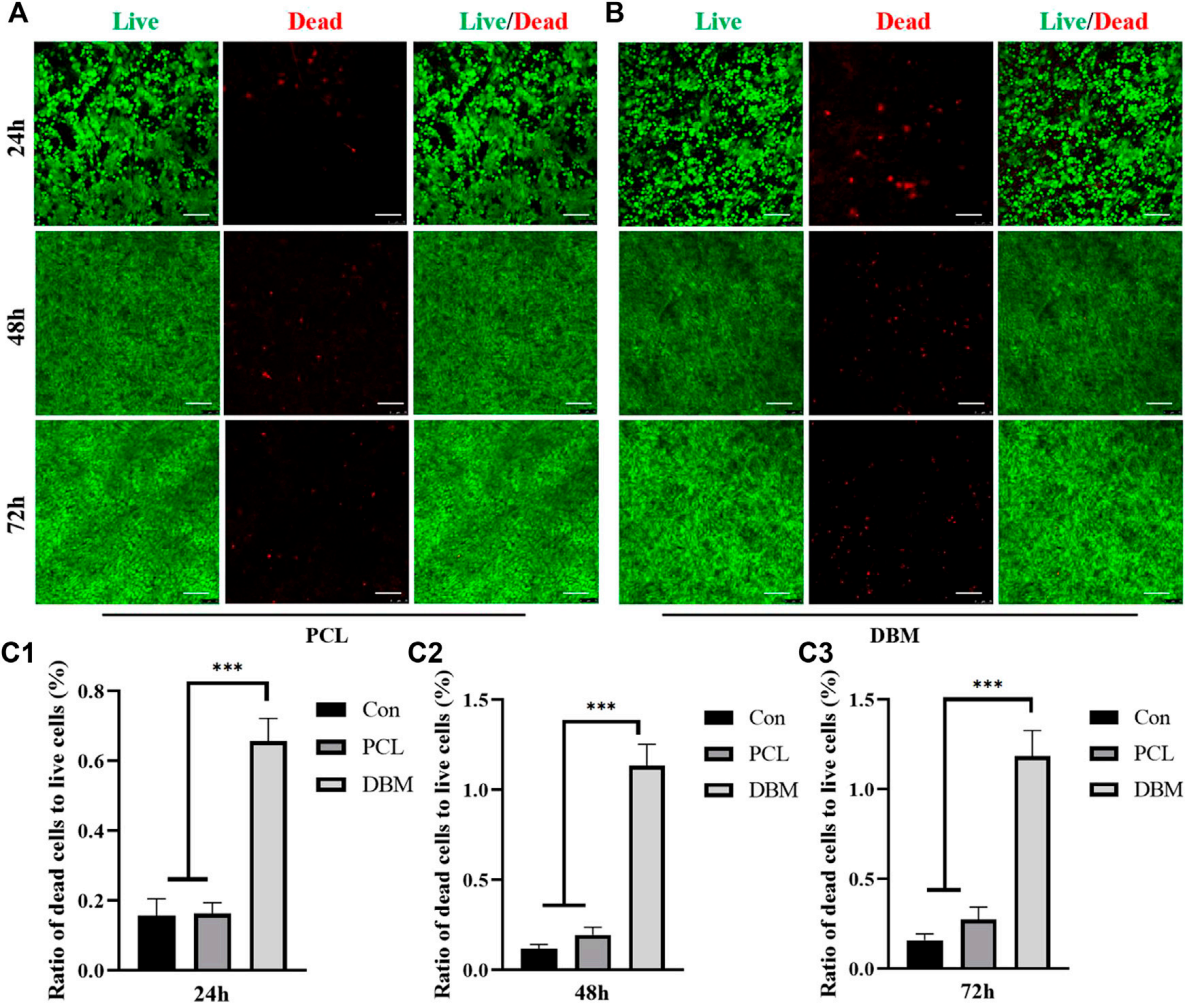
Cytotoxicity evaluation of the frameworks to ECG. **(A)** Live/dead staining of ECG in PCL leach solution for 24, 48, and 72 h; **(B)** Live/dead staining of ECG in DBM leach solution for 24, 48, and 72 h; Semi-quantitative analysis for the ratio of dead cells to live cells at 24h **(C1)**, 48h **(C2)**, and 72h **(C3)**. Statistical significance: ∗∗∗*p* < 0.001. Scale bar: 100μm.

The biocompatibility was then evaluated by loading ECG into the frameworks. Similar to our previous reports, cartilage sheets cultured *in vitro* for 5 days presented soft fragile membranes that could be easily collected, minced into gel form (Figures 2A,B) while maintaining good cellular viability (Figures 2C,D), and then loaded into the frameworks (Figure 2E). SEM showed that ECG adhered well to the frameworks and completely covered the frameworks after 3 days of *in vitro* culture owing to the abundant ECM production (Figures 2F,G). The quantification analysis revealed that the ECG loading efficiencies in the two frameworks were both higher than 90% with no statistical difference (Figure 2H), indicating good cytocompatibility for both frameworks. Cytotoxicity evaluation showed that cartilage sheets survived well in the leach solutions of both frameworks with visible cell proliferation over time (Figures 3A,B). Notably, few dead cells were observed in the group treated with PCL leach solution (no significant difference with the DMEM control group, Supplementary Figure S1), while some dead cells were found in the group treated with DBM leach solution (Figure 3C). This indicates the higher cytotoxicity of the DBM framework compared with the PCL framework, which might be related to higher endotoxin residue in the DBM framework (Supplementary Figure S2).

### 3.2 *In Vivo* Inflammatory Response Triggered by Polycaprolactone and Decalcified Bone Matrix Frameworks

**FIGURE 4:**
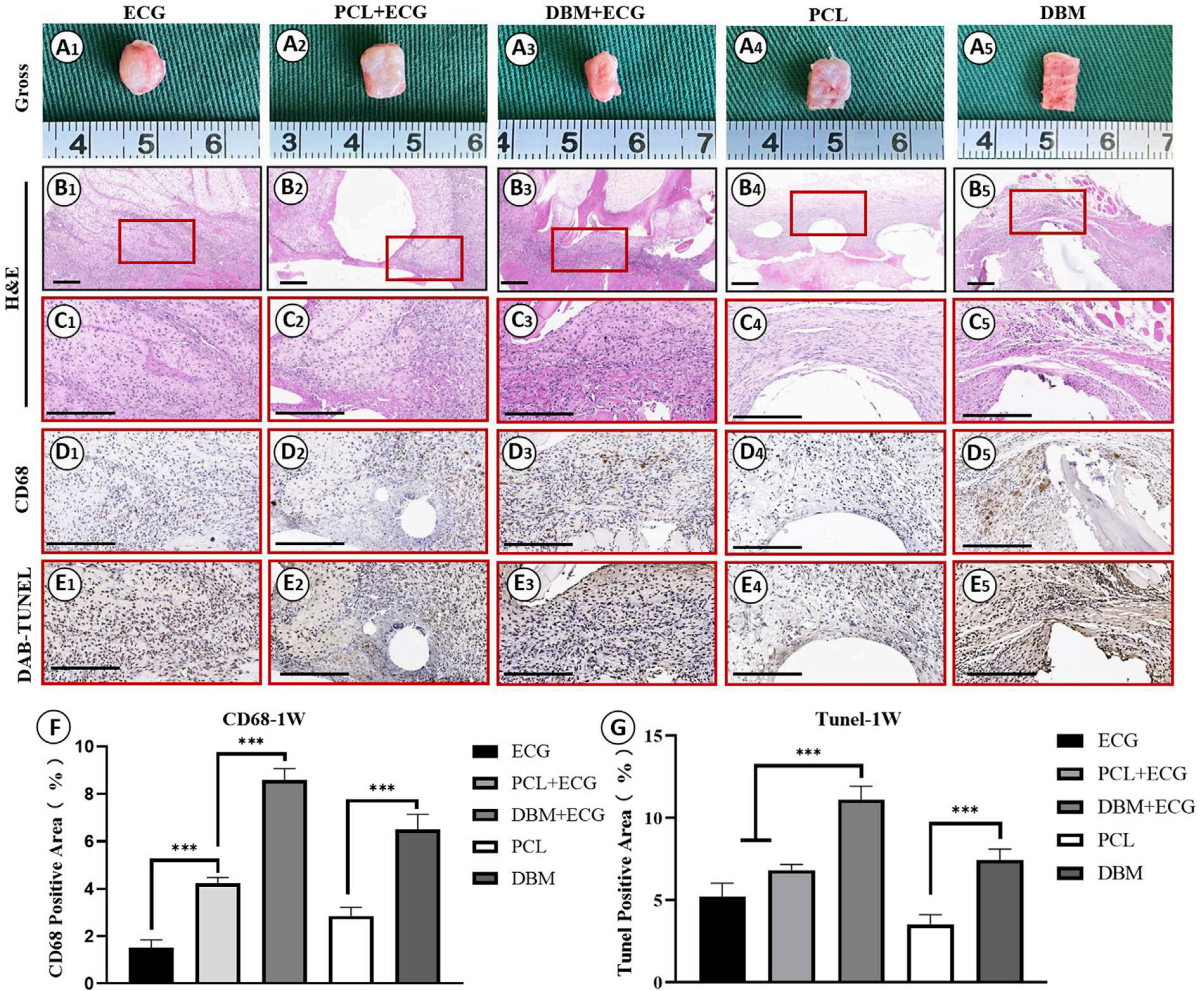
Inflammatory response evaluations of ECG, frameworks, and framework-ECG constructions after 1 week of subcutaneous implantation: Gross observation **(A1–AA)**; H&E staining with different magnification **(B1–B5, C1–C5)**; CD68 immunohistochemical staining **(D1–D5)**; DAB-TUNEL immunohistochemical staining **(E1–E5)**; Semi-quantitative analysis of the CD68 **(F)** and DAB-TUNEL **(G)** positive area (%). Statistical significance: ∗*p* < 0.05, ∗∗*p* < 0.01, ∗∗∗*p* < 0.001. Scale bar: 200 μm.

**FIGURE 5:**
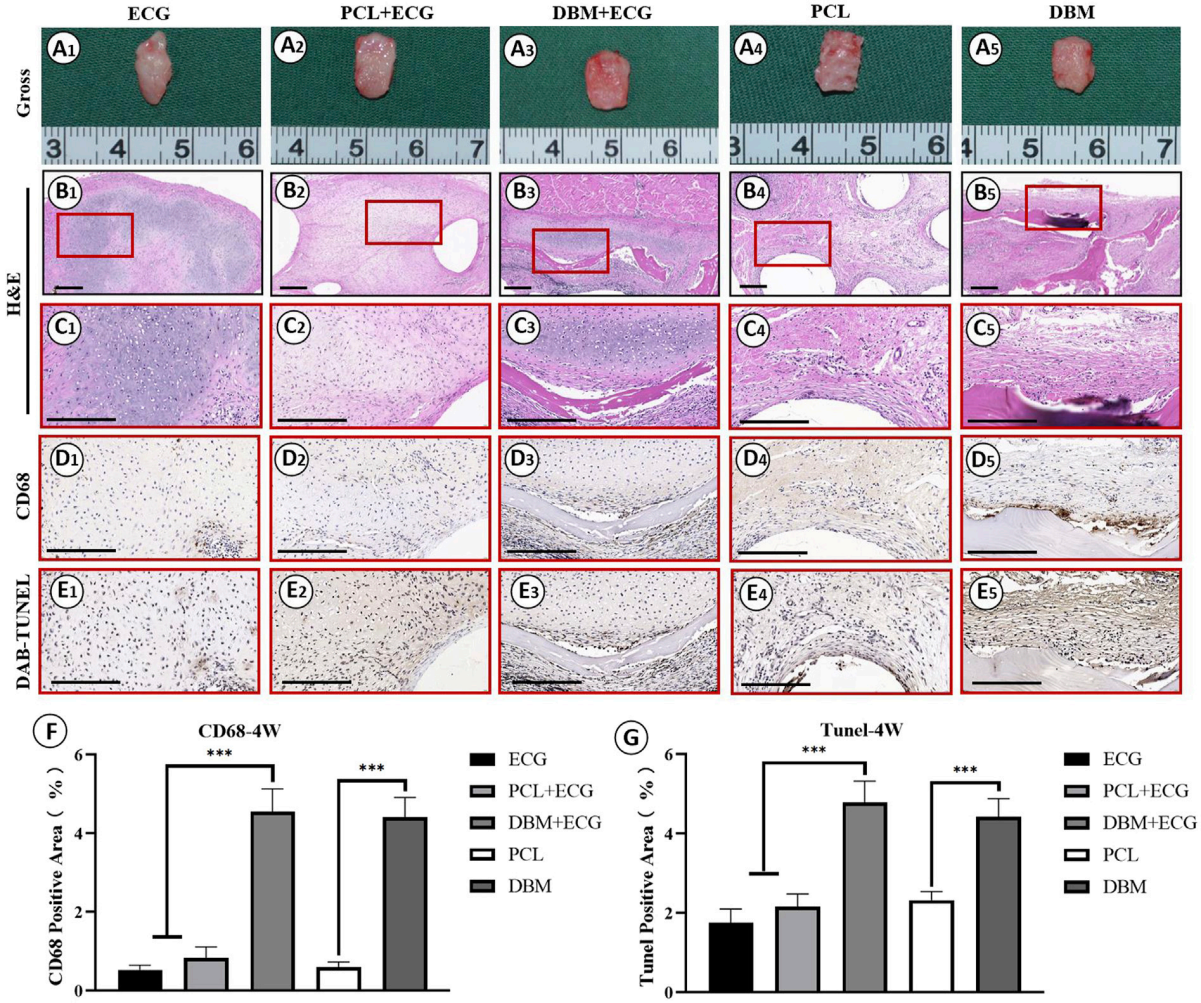
Inflammatory response evaluations of ECG, frameworks, and framework-ECG constructions after 4 week of subcutaneous implantation: Gross observation **(A1–A5)**; H&E staining with different magnification (**B1–B5, C1–C5)**; CD68 immunohistochemical staining **(D1–D5)**; DAB-TUNEL immunohistochemical staining **(E1–E5)**; Semi-quantitative analysis of the CD68 **(F)** and DAB-TUNEL **(G)** positive area (%). Statistical significance: ∗*p* < 0.05, ∗∗*p* < 0.01, ∗∗∗*p* < 0.001. Scale bar: 200 μm.

*In vivo* inflammatory responses were evaluated to predict the feasibility of cartilage regeneration using the PCL-ECG constructs. The observations after 1 week show that all samples were easily extracted from the implantation sites without obvious adhesion to peripheral tissues (Figure 4A), suggesting relatively low inflammatory responses in all groups. H&E and CD68 immunohistochemical staining showed that the inflammatory responses for both the PCL-ECG and DBM-ECG groups were much stronger than that for the ECG group, indicating that both the PCL and DBM frameworks showed some immunogenicity. This was further supported by the inflammatory responses triggered by the implantation of frameworks without ECG (Figures 4B–D). TUNEL immunohistochemical staining also confirmed that more apoptotic cells caused by inflammatory response were found for the groups containing frameworks than for the ECG group (Figure 4E). It was worth noting that the PCL framework triggered a milder inflammatory response with less cell apoptosis than the DBM framework (Figures 4D,E), which was further confirmed by a semiquantitative analysis (Figures 4F,G), indicating that the PCL framework had lower immunogenicity than the DBM framework. As anticipated, the intensity of the inflammatory responses at 4 weeks was lower than those for the samples after 1 week, with less cell apoptosis in all groups (Figures 5A–E). Semiquantitative analysis further revealed that both the ECG and PCL-ECG groups presented minimal inflammatory infiltration and cell apoptosis (with no significant difference), while the DBM framework still exhibited higher levels of inflammatory response and cell apoptosis (Figures 5F,G). This indicates that the PCL framework is more suitable for supporting cartilage regeneration of ECG than the DBM framework in terms of immunogenicity.

### 3.3 *In Vitro* Macrophage M1 Polarization Triggered by Polycaprolactone and Decalcified Bone Matrix Frameworks

**FIGURE 6:**
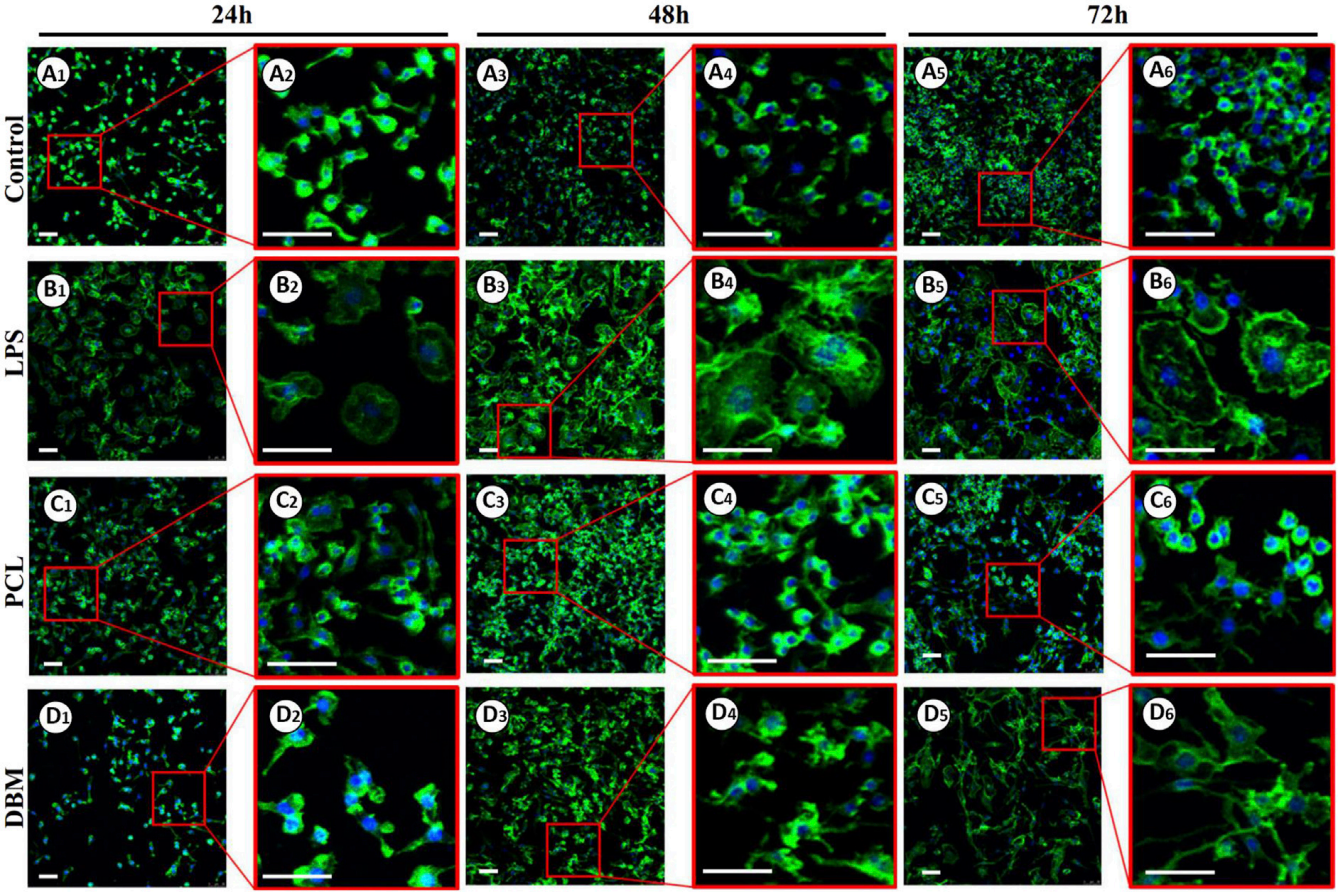
Phalloidine immunofluorescence staining of RAW 264.7 cells treated by leach solutions of PCL and DBM frameworks: Negative control cultured in DMEM **(A1–A6)**; Positive control activated by LPS **(B1–B6)**; RAW 264.7 cells treated by PCL leach solution **(C1–C6)**; RAW 264.7 cells treated by DBM leach solution **(D1–D6)**. Scale bar: 100 μm.

**FIGURE 7:**
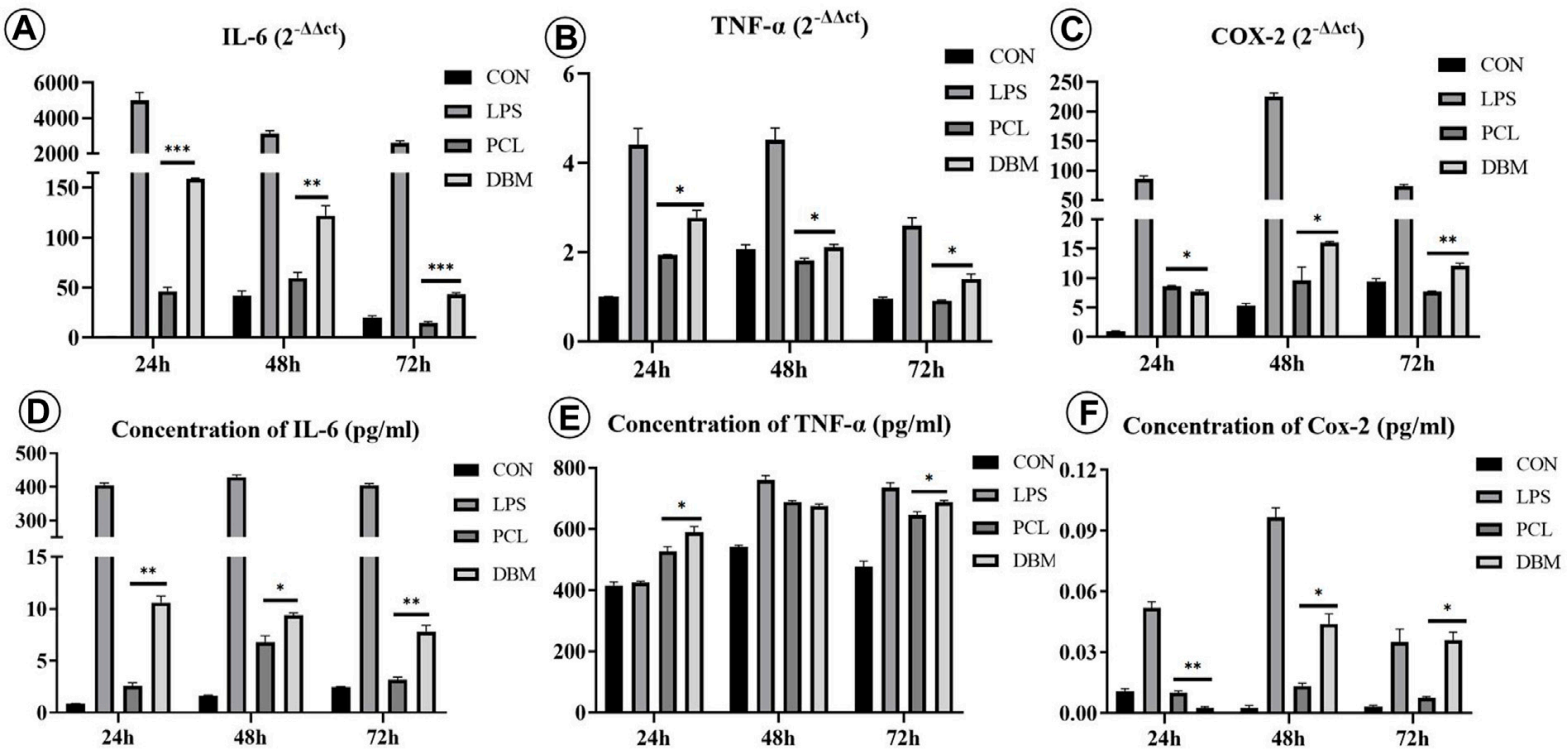
Gene expression and cytokine secretion related to M1 polarization of RAW 264.7 cells treated by leach solutions of PCL and DBM frameworks: **(A–C)** qPCR analysis of M1 polarization-related genes IL-6 **(A)**, TNF-α **(B)**, and COX-2 **(C)**; **(D–F)** ELISA quantification of M1 polarization-related cytokines IL-6 **(D)**, TNF-α **(E)**, and COX-2 **(F)**. Statistical significance: *p < 0.05, **p < 0.01, ***p < 0.001.

An *in vitro* macrophage M1 polarization experiment was conducted to further evaluate the framework immunogenicity. As shown in Figure 6, RAW 264.7 cells cultured in DMEM presented relatively uniform morphology with small round cortical actin rings (Figure 6A), while the cells treated with LPS exhibited typical M1 polarization morphology with large dendritic cortical actin rings (Figure 6B). Cells cultured in the leach solution of the PCL framework showed minor morphology changes essentially maintaining small round cortical actin rings (Figure 6C), while cells cultured in the leach solution of the DBM framework presented a discernible morphology change, exhibiting relatively large and irregular cortical actin rings (Figure 6D). The results of qPCR and ELISA were consistent with the cell morphology changes, further confirming that the expressions of M1 polarization related cytokines (IL-6, COX-2, and TNF-α) in the PCL group were significantly lower than those for the DBM group in terms of both gene and protein levels (Figure 7). These results indicate that the leach solution of the PCL framework triggered milder M1 polarization of macrophages than the leach solution of the DBM framework, suggesting that the PCL framework showed lower immunogenicity than the DBM framework.

### 3.4 *In Vivo* Cartilage Regeneration of the Framework–Engineered Cartilage Gel Constructs

**FIGURE 8:**
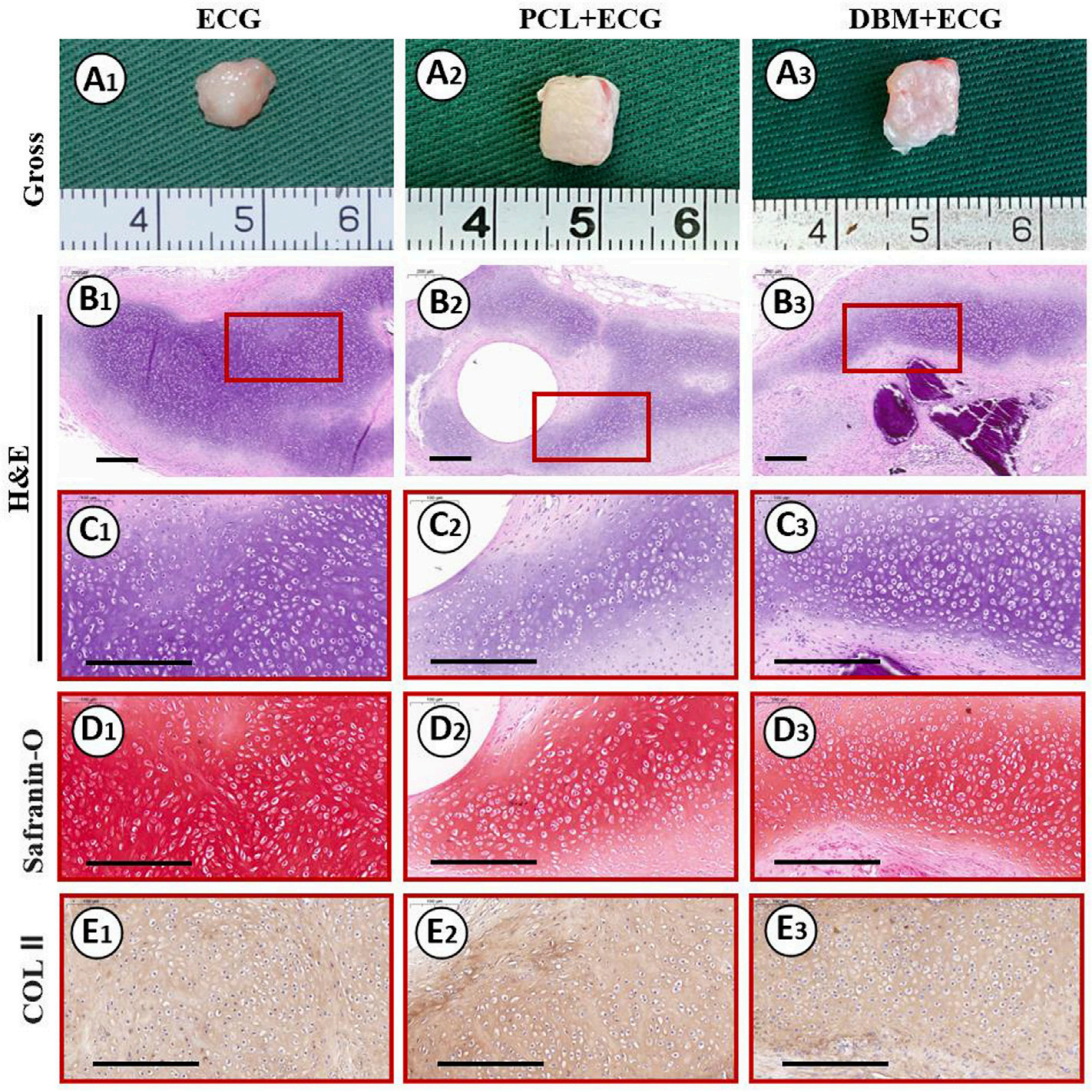
Gross view and histological examinations of the regenerated cartilage in ECG, PCL-ECG, and DBM-ECG groups after 8 weeks of subcutaneous implantation: Gross observation **(A1–A3)**; H&E staining with different magnification **(B1–B3, C1–C3)**; Safranin-O staining **(D1–D3)**; COL II immunohistochemical staining **(E1–E3)**. Scale bar: 200 μm.

**FIGURE 9:**
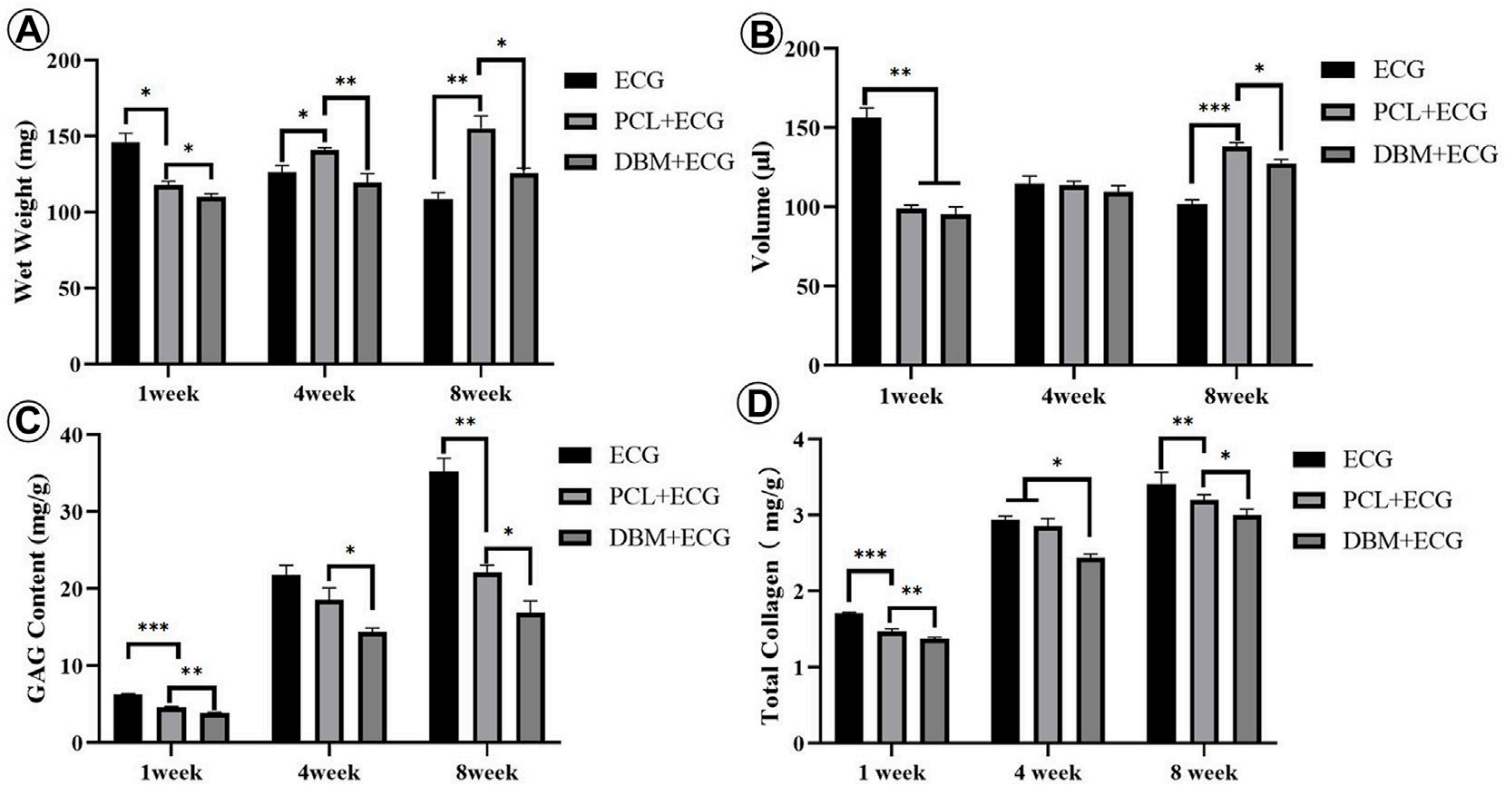
Quantitative evaluations of the regenerated cartilage in ECG, PCL-ECG, and DBM-ECG groups after 1, 4, and 8 weeks of subcutaneous implantation: **(A)** wet weight, **(B)** volume, **(C)** total glycosaminoglycan (GAG) content, and **(D)** total collagen content. Statistical significance: ∗p < 0.05, ∗∗p < 0.01, ∗∗∗p < 0.001.

The *in vivo* cartilage regeneration performance was analyzed to evaluate the clinical translational potential of the PCL-ECG strategy. Gross observation showed that the regenerated cartilage in all groups gradually matured as evidenced by a reddish appearance at 1 week to an ivory appearance at 8 weeks (Figures 4A, 5A, 8A). The samples in the PCL-ECG group essentially maintained their original shape and size, showing relatively regular cuboids, while the samples in the DBM-ECG group showed slight deformation with an irregular cuboid shape (Figure 8A). As anticipated, the samples in the ECG group showed an irregular shape due to lack of a supporting framework (Figure 8A). Histological analysis revealed that the samples in all groups formed mature cartilage-like tissue with typical lacuna structures and abundant cartilage-specific ECM deposition evidenced by strong positive staining of safranin-O and collagen II (Figures 8B–E). The quantitative analysis showed that the GAG and total collagen content of the regenerated cartilage in all groups showed a gradually increasing trend, indicating the gradual maturation of neo-cartilage, which was further supported by the gradually increasing wet weights and volumes observed for both the PCL-ECG and DBM-ECG groups. Notably, the wet weight and volume of the ECG group decreased over time, which might be related to stress induced absorption due to lack of a supporting framework (Supplementary Figure S3). In addition, it is worth noting that all of the quantitative data for the PCL-ECG group were higher than those for the DBM-ECG group (Figure 9), indicating relatively higher cartilage yield in the PCL-ECG group. These results suggest excellent clinical translation potential for the PCL-ECG cartilage regeneration strategy.

## 4 Discussion

Although the feasibility of the steel-reinforced concrete cartilage regeneration model with ECG inoculated into DBM frameworks was confirmed by our previous study (Ci et al., 2021), the disadvantages of DBM hindered the further clinical translation of this technology. The current study shows that a 3D-printed PCL framework exhibits better biocompatibility and immunogenicity than the DBM framework. More importantly, after 8 weeks of subcutaneous implantation in large animals, the PCL-ECG constructs successfully regenerated mature cartilage and essentially maintained their original shape, which indicated that the PCL framework could serve as a promising framework to replace DBM in supporting 3D cartilage regeneration of ECG. As an FDA-approved 3D-printable biomaterial, PCL can be prepared in a variety of 3D shapes with controlled mechanical strength by computer-assisted design and thus has excellent potential for clinical translation.

The suitability of the 3D-printed PCL frameworks for ECG loading was the primary concern of this study. To provide a solid mechanical support while ensuring high loading efficiency, a double-layered PCL framework structure was designed. The top layer—the supporting and loading layer—was printed with thick strands and large pores to provide sufficient mechanical strength and ECG loading space, while the bottom layer was printed as a dense grid with thin strands and small pores to prevent ECG loss. The validity of this framework design was fully confirmed by the findings that the PCL framework exhibited high mechanical strength and could integrate well with ECG with high loading efficiency. Notably, although there are still no unified criteria for the mechanical strength of the frameworks, the strong and controllable mechanical properties mean that the framework provided sufficient mechanical support for the early implantation of the constructs, particularly for the repair of cartilage defects in the subcutaneous environment, and to make necessary adjustments according to the specific situation of different patients. Furthermore, the PCL framework could be filled with ECG, which effectively reduced the exposure of the PCL framework. A cell viability assay confirmed that the PCL leach solution had no negative effects on the survival and proliferation of the cartilage sheets. However, in the control group, the DBM leach solution showed clear cytotoxicity and caused a small amount of apoptosis in the cartilage sheets, which is attributed to endotoxin residues in the DBM framework. These results suggest that the 3D-printed PCL framework could serve as an ideal supporting material for ECG loading in terms of mechanical strength, loading efficiency, and biocompatibility.

Inflammatory responses in large animals are important factors that affects cartilage regeneration and its clinical translation (Padmanabhan and Kyriakides, 2015; Koh et al., 2020). Therefore, the immunogenicity of the PCL framework was evaluated. The results show that the PCL framework caused a milder inflammatory response with less macrophage infiltration and chondrocyte apoptosis than the DBM framework. *In vitro* results further confirmed that the M1 polarization of macrophages activated by the PCL framework was significantly weaker than that caused by the DBM framework. PCL is an FDA-approved polyester biomaterial (Yadav et al., 2022) that has good biocompatibility and a relatively slow degradation rate with neutral and nontoxic degradation products (Panigrahy and Rath, 2018; Backes et al., 2021; Bazgir et al., 2021), which may explain the low immunogenicity shown in this study. Although DBM is a natural biomaterial, during the production process, certain harmful bioactive components such as endotoxin and xenogenic protein are unavoidably retained to preserve the bioactivity of DBM (Shi et al., 2018; Amirazad et al., 2022) (Supplementary Figure S2), which might be why DBM triggered a more severe inflammatory response. These results suggest that the 3D-printed PCL framework is a better support for ECG loading than the DBM framework in terms of immunogenicity.

The ability of the PCL-ECG constructs to regenerate high-quality cartilage is the final criterion for evaluating the clinical potential of the strategy. The results show that the PCL-ECG constructs successfully regenerated mature cartilage with typical lacuna and cartilage-specific ECM deposition. Furthermore, the regenerated cartilage in the PCL-ECG group exhibited better shape maintenance with a higher cartilage-specific matrix content than that of the DBM-ECG group. The better shape maintenance exhibited by the PCL-ECG constructs is attributed to the appropriate mechanical strength (Olubamiji et al., 2016) and homogeneous structure throughout the PCL framework. In contrast, the DBM framework had relatively low mechanical strength and a heterogeneous structure, which led to poorer shape maintenance. In addition, the biocompatibility and low immunogenicity of the PCL framework ensured satisfactory cartilage regeneration in the PCL-ECG group, while the observed cytotoxicity and immunogenicity of the DBM framework led to relatively poorer cartilage regeneration in the DBM-ECG group. Notably, the ECG group with no framework triggered the mildest inflammatory reaction and achieved the optimal cartilage regeneration. Nevertheless, the uncontrolled shape and visible absorption, likely caused by the lack of mechanical support, would greatly limit its clinical application in cartilage defects with specialized shape. These results indicate that the 3D-printed PCL framework provided a stable support for ECG cartilage regeneration with satisfactory shape maintenance and cartilage quality.

## 5 Conclusion

A novel strategy for 3D cartilage regeneration based on a 3D-printed PCL framework and ECG was demonstrated. The PCL framework exhibited controllable 3D shape, homogeneous structure, appropriate mechanical strength, high loading efficiency, good biocompatibility, and low immunogenicity and successfully supported mature cartilage regeneration of ECG with satisfactory shape maintenance and cartilage quality. Although further investigations are required—for example, to optimize of the 3D-printing parameters for the PCL framework, determine the feasibility of regenerating cartilage with complex 3D shapes, and repair cartilage defects with complex 3D shapes in a large animal model—the current study demonstrates a novel strategy for ECG-based 3D cartilage regeneration for the repair of craniofacial cartilage defects.

## Data Availability

The original contributions presented in the study are included in the article/Supplementary Material, further inquiries can be directed to the corresponding authors.
