# Corrigendum: Three-Dimensional Cartilage Regeneration Using Engineered Cartilage Gel With a 3D-Printed Polycaprolactone Framework

**DOI:** 10.3389/fbioe.2022.951730

**Published:** 2022-06-14

**Authors:** Gaoyang Wu, Lixing Lu, Zheng Ci, Yahui Wang, Runjie Shi, Guangdong Zhou, Shengli Li

**Affiliations:** ^1^ Department of Plastic and Reconstructive Surgery, Shanghai Ninth People’s Hospital, Shanghai Jiao Tong University School of Medicine, Shanghai, China; ^2^ Department of Otorhinolaryngology Head and Neck Surgery, Shanghai Ninth People’s Hospital, Shanghai Jiao Tong University School of Medicine, Shanghai, China; ^3^ National Tissue Engineering Center of China, Shanghai, China; ^4^ Research Institute of Plastic Surgery, Weifang Medical University, Weifang, China; ^5^ Shanghai Key Laboratory of Translational Medicine on Ear and Nose Diseases, Ear Institute Shanghai Jiao Tong University School of Medicine, Shanghai, China

**Keywords:** 3D cartilage regeneration, engineered cartilage gel, Polycaprolactone, inflammatory response, tissue engineering

In the published article, the fourth and fifth affiliations were inverted, the fourth affiliation should be “^4^Research Institute of Plastic Surgery, Weifang Medical University, Weifang, China,” and the fifth affiliation should be “^5^Shanghai Key Laboratory of Translational Medicine on Ear and Nose Diseases, Ear Institute Shanghai Jiao Tong University School of Medicine, Shanghai, China.”

The authors apologize for this error and state that this does not change the scientific conclusions of the article in any way. The original article has been updated.

