# Supplementary material for: Three-Dimensional Cartilage Regeneration Using Engineered Cartilage Gel With a 3D-Printed Polycaprolactone Framework
Source: Front Bioeng Biotechnol. 2022 May 24;10:871508. doi: 10.3389/fbioe.2022.871508 (PMC9171075; doi:10.3389/fbioe.2022.871508)
Supplement: Supplementary file 1 [file DataSheet1.PDF]

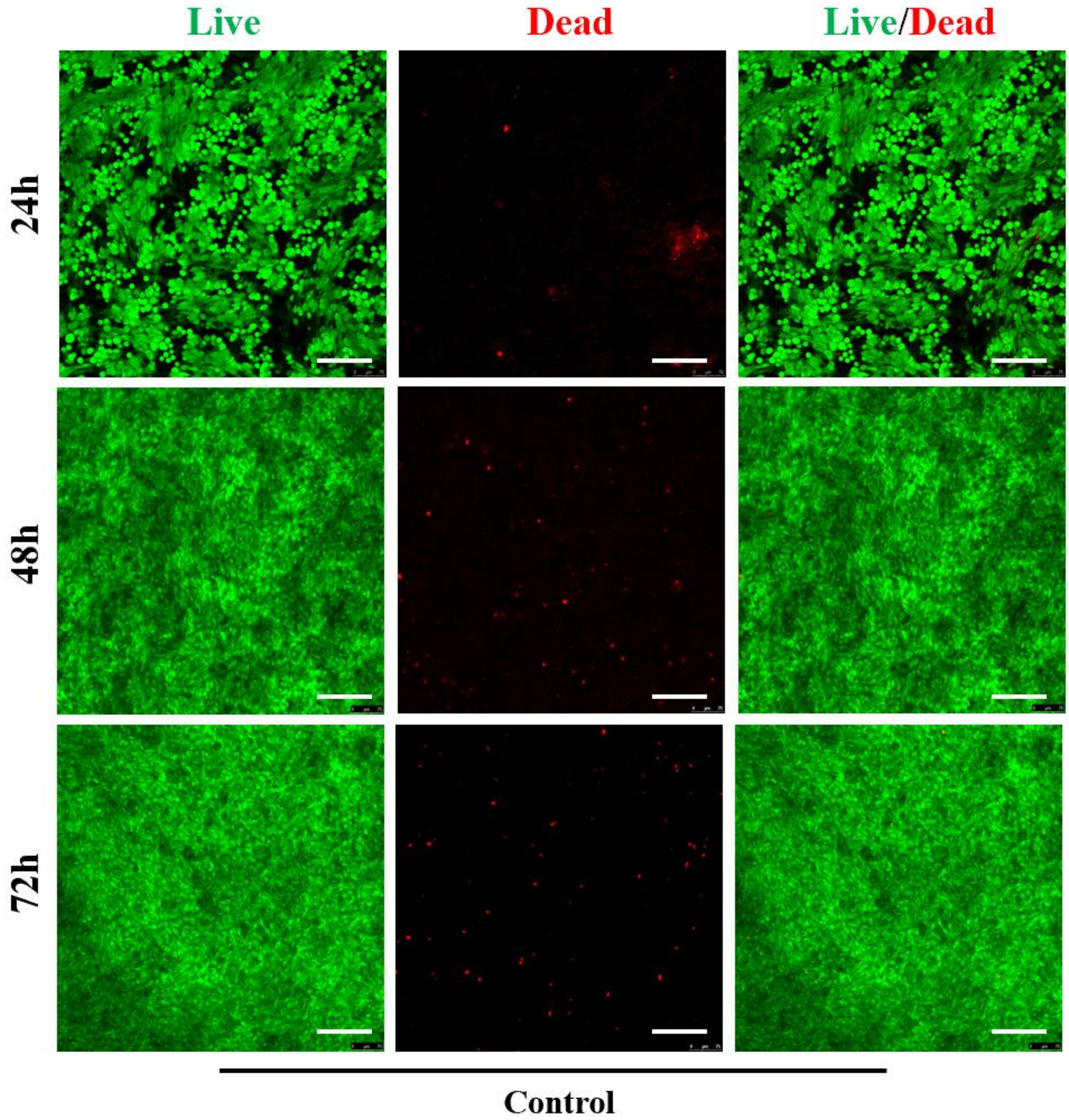

Supplementary Figure 1. Live/dead staining of ECG in DMEM for 24, 48, and 72 h. Scale bar: 100μm.

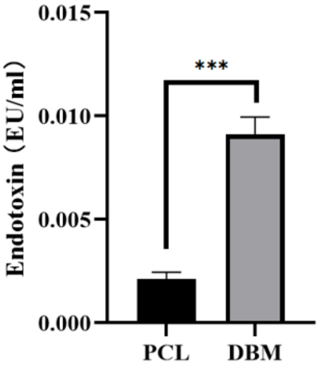

Supplementary figure 2. Endotoxin concentrations in PCL and DBM leach solutions.

Statistical significance: ***$p < 0.001$.

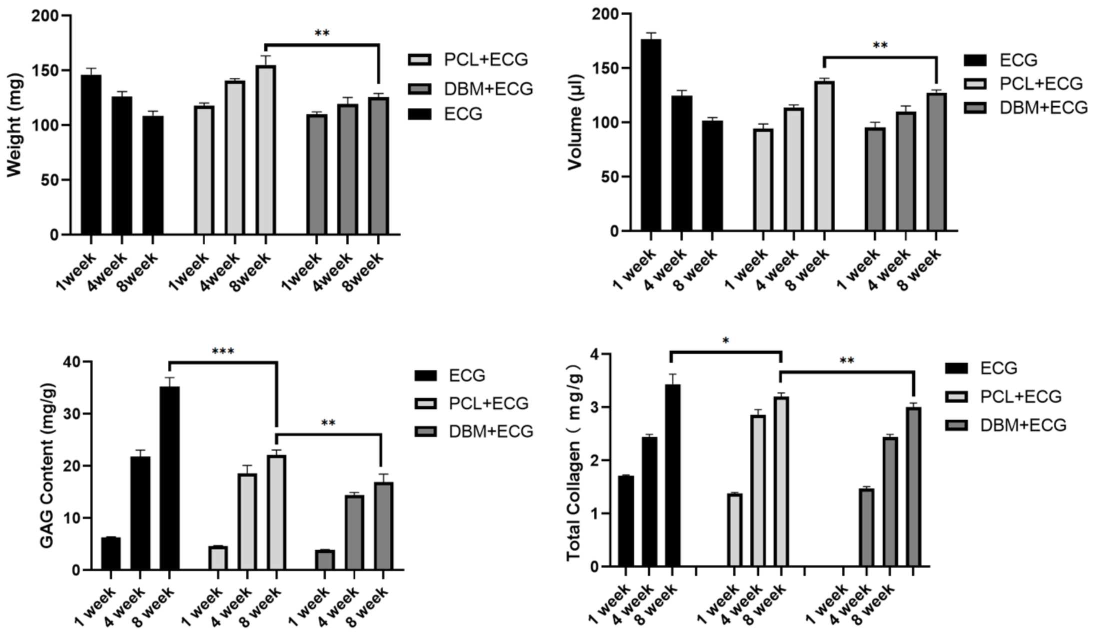

Supplementary Figure 3. Cartilage regeneration process in ECG, PCL-ECG, and DBM-ECG groups after subcutaneous implantation: (A) Wet weight; (B) Volume; (C) Total glycosaminoglycan (GAG) content; (D) Total collagen content. Statistical significance: $*p < 0.05$, $**p < 0.01$, $***p < 0.001$.
